# Internal hernia through a mesoappendiceal defect causing gangrenous small bowel obstruction and gangrenous appendix: a case report and literature overview

**DOI:** 10.1097/RC9.0000000000001010

**Published:** 2026-10-06

**Authors:** Eyob Shiferaw Woldekidan, Sewnet Ejigu Mekonnen, Ermias Gebremeskel Gebreslassie, Natnael Abera Asrat, Fiseha Teshome Kebede

**Affiliations:** a Jinka General Hospital, Southern Ethiopia Regional Health Bureau, Jinka, Ethiopia; b Yejubie Primary Hospital, Amhara Regional Health Bureau, Bahrdar, Ethiopia; c Yirba Primary Hospital, Sidama Regional Health Bureau, Hawassa, Ethiopia

**Keywords:** case report, internal hernia, mesoappendix, small bowel obstruction

## Abstract

**Background:**

An internal hernia is a cause of small-bowel obstruction and may lead to bowel ischemia and strangulation if not diagnosed early. Herniation through a defect in the mesoappendix is an exceptionally uncommon type of internal hernia, with only a few cases reported in the literature. Because the clinical presentation is nonspecific, preoperative diagnosis is difficult and most cases are identified during surgery.

**Case presentation:**

We report a case of a 42 years old female patient who presented with symptoms and signs of small bowel obstruction and generalized peritonitis, whose cause was diagnosed intraoperatively as an internal hernia through the mesoappendix. Intraoperative findings revealed a gangrenous segment of ileum herniated through a defect in the mesoappendix, and the distal shaft and tip of the appendix were also gangrenous. She was managed with *en bloc* resection, ileostomy, and appendectomy. Postoperative management included antibiotic therapy and wound care. The patient recovered uneventfully and was discharged home in stable condition.

**Discussion:**

Internal herniation through a mesoappendiceal defect is an extremely rare cause of small-bowel obstruction and poses a diagnostic challenge because its clinical presentation is nonspecific in most cases; definitive diagnosis is made intra-operatively. Surgical exploration remains critical for both diagnosis and management of this potentially life-threatening condition.

**Conclusion:**

Small-bowel obstruction secondary to internal herniation through a mesoappendiceal defect should be considered in the differential diagnosis of patients presenting with small-bowel obstruction.

## Introduction

Small bowel obstruction (SBO) is a common surgical emergency, accounting for a significant proportion of acute abdominal admissions worldwide^[^1^]^. The most frequent causes include postoperative adhesions, external hernias, and malignancy; however, internal hernias represent a rare etiology, accounting for approximately 0.6–5.8% of all cases of intestinal obstruction^[^2–4^]^. Despite their rarity, internal hernias are clinically important due to their propensity to cause closed-loop obstruction and bowel strangulation, which may lead to significant morbidity and mortality if not promptly diagnosed and managed^[^2,5^]^.

HIGHLIGHTS

- An internal hernia through a mesoappendiceal defect is an extremely rare cause of small-bowel obstruction.
- Preoperative diagnosis is challenging because clinical findings are nonspecific.
- Although internal hernia is rare, it should be considered in patients with bowel obstruction.
- Early surgical intervention prevents bowel ischemia and strangulation.
- Definitive management involves reduction of the herniated bowel if viable, or resection if gangrenous, along with appendectomy.

Internal hernia defects may be congenital or acquired, with acquired causes increasingly reported in association with prior abdominal surgery, inflammation, or mesenteric defects^[^6,7^]^. Recent literature continues to highlight the diagnostic challenge posed by internal hernias due to their nonspecific clinical presentation and variable imaging findings^[^7,8^]^

Herniation through defects in the mesoappendix is exceedingly rare and sparsely reported in the literature^[^9^]^. Defects in the mesoappendix may arise secondary to prior inflammation, such as appendicitis, ischemic changes, or surgical manipulation, creating a pathway for small bowel loops to herniate^[^9,10^]^. This can result in mechanical obstruction and, in severe cases, vascular compromise leading to bowel ischemia. Due to the rarity of this condition and the absence of specific clinical or radiological features, diagnosis is often made intraoperatively^[^4,9^]^.

Case reports and reviews emphasize that internal hernias should be considered in patients presenting with SBO, particularly in the absence of previous abdominal surgery, as delayed diagnosis may lead to increased morbidity and bowel loss^[^2,4^]^.

In this case report, we present a case of SBO secondary to internal herniation through a defect in the mesoappendix, highlighting the diagnostic challenges and surgical management. Although different types of internal hernias occur, to our knowledge, herniation through the mesoappendix has been reported only seven times in the literature (all seven references). This case report has been reported in accordance with the Surgical CAse REport (SCARE) guidelines.

## Case presentation

We report the case of a 42 years old female patient who presented to our hospital with a complaint of crampy abdominal pain of 2 days’ duration. The abdominal pain initially was periumbilical, which later became generalized. The pain was associated with repeated episodes of vomiting, initially containing recently ingested food particles and later becoming bilious. She also noticed progressive abdominal distension, and an inability to pass both feces and flatus of the same duration. There was no history of trauma, fever, hematemesis, melena, or jaundice. There was no history of similar previous episodes or abdominal surgery. Past medical history was unremarkable for diabetes mellitus, hypertension, or chronic illnesses.

On examination, vital signs were as follows: blood pressure (BP) 128/78, pulse rate (PR) 104 beats per minute, temperature 36.5 °C, and oxygen saturation 94%. On abdominal examination, the abdomen was markedly distended and did not move with respiration. There was generalized tenderness associated with guarding, rigidity, and rebound tenderness, consistent with generalized peritonitis. Bowel sounds were hypoactive. Digital rectal examination revealed scant stool in the rectum, with no evidence of blood or a palpable rectal mass.

The following investigations were done: complete blood count; WBC 8.28 × 10^3^/µL; Hgb 12 g/dL; Hct 34.8%; platelets 215 **×** 10^3^/µL; creatinine 0.68 mg/dL; Na + 134 mmol/L; K + 3.0 mmol/L.

As the patient presented with the cardinal clinical features of small-bowel obstruction and generalized peritonitis, both of which are indications for emergency surgical intervention, we proceeded directly to an exploratory laparotomy without additional imaging to avoid delaying definitive management.

The patient underwent exploratory laparotomy with an impression of generalized peritonitis secondary to suspected gangrenous small-bowel volvulus, and intra-operatively there was an approximately 30 cm gangrenous segment of ileum within an internal hernia through a defect in the mesoappendix (Fig. 1) and approximately 400 mL of hemorrhagic fluid. The distal shaft and tip of the appendix were gangrenous, and the base was viable. Only 2 cm of ileum from the ileocecal valve was viable. En bloc resection of the gangrenous ileum, lavage with warm saline, and ileostomy were performed because of the massive fecaloma in the colon. The distal viable stump of ileum was closed. Appendectomy was performed and the defect was removed with the specimen. Then the patient was admitted to the surgical ward on intravenous antibiotics (Ceftriaxone 1000 mg IV BID, Metronidazole 500 mg IV TID) and wound care for a surgical site infection. The antibiotics were given for 8 days and the regimen remained unchanged. She did not require higher-level care such as ICU or HDU. The patient was discharged home after 8 days of hospitalization.

**Figure 1.**
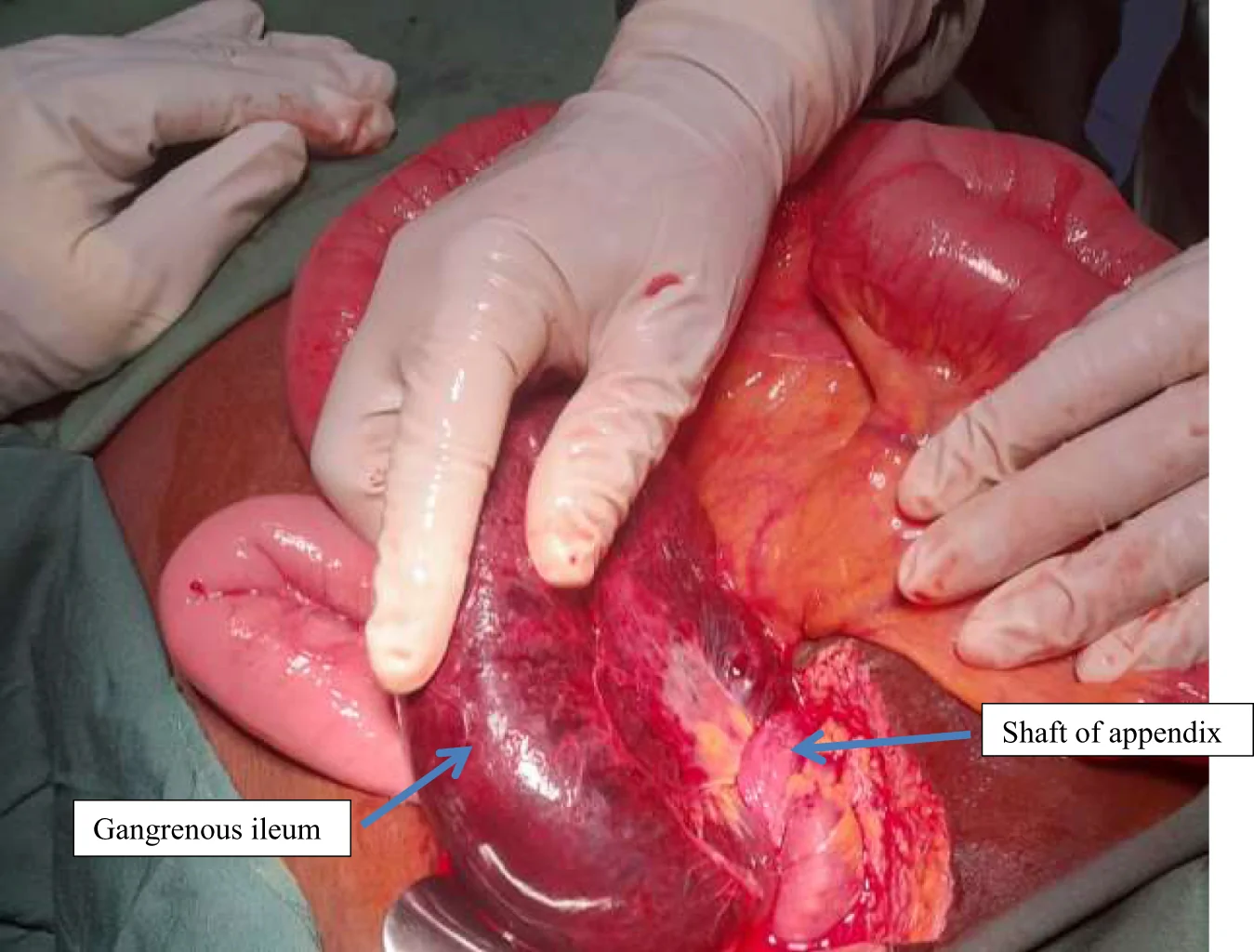
Intraoperative photograph showing a loop of gangrenous ileum herniated through a defect in the mesoappendix.

The patient was followed at the surgical referral clinic, and during follow-up, the patient remained clinically well, with no stoma-related complications, hospital readmissions, or recurrent intestinal obstruction. Ileostomy reversal was performed 8 months after the initial surgery. The delay in restoration of bowel continuity was primarily due to financial constraints. Readiness for ileostomy reversal was assessed based on the patient’s clinical recovery and nutritional status. Specifically, reversal was performed after confirming that the patient had a normal serum albumin level and normal hemoglobin concentration, indicating adequate nutritional and hematologic status for surgery. Based on the intraoperative findings, including adhesions involving the ileocecal region, and to achieve a tension-free, well-vascularized anastomosis and to facilitate safe restoration of bowel continuity, an ileotransverse end-to-side anastomosis was performed. After restoration of bowel continuity, the patient had an uncomplicated postoperative recovery. There were no anastomotic or surgical site complications, and she was discharged on postoperative day 7. During follow-up, she remained clinically well without any reported complications.

The patient expressed happiness and gratitude for the treatment she received, particularly after she improved and was discharged home. She reported significant relief from her initial anxiety and was satisfied with the care provided and the outcome of the treatment.

## Discussion

Internal hernias are an uncommon but important cause of small-bowel obstruction (SBO), accounting for a small proportion of cases but carrying a high risk of strangulation and bowel ischemia if diagnosis is delayed^[^4^]^. Trans-mesoappendicular hernias are an infrequently reported subtype of internal hernia, with limited cases described in the existing literature^[^4,9^]^.

The pathogenesis of internal herniation through the mesoappendix remains poorly understood. However, both congenital and acquired mechanisms have been proposed. Congenital defects may arise from developmental abnormalities of the mesoappendix, while acquired defects may result from inflammation, appendicitis, trauma, or prior surgical manipulation^[^9–11^]^. Some reports have suggested that anatomical variations, such as an unusually long appendix, may predispose to the formation of a defect and subsequent herniation^[^11^]^.

Clinically, patients with trans-mesoappendicular hernia typically present with features of acute SBO, including abdominal pain, vomiting, distension, and constipation^[^9^]^. However, these symptoms are nonspecific and indistinguishable from other causes of SBO, making preoperative diagnosis challenging. Imaging modalities such as computed tomography (CT) may suggest an internal hernia by demonstrating clustered bowel loops, transition points, or closed-loop obstruction, but definitive diagnosis is often made intra-operatively^[^4,12^]^. In the case reported by Harvitkar *et al*, CT demonstrated features of closed-loop obstruction, but the exact etiology was only confirmed during laparoscopy^[^4^]^.

A notable feature of many reported cases is the absence of prior abdominal surgery, which should raise suspicion for an internal hernia as a cause of obstruction; however, cases have also been reported in patients with prior surgical history, indicating that this condition should not be excluded solely on that basis^[^4,9,11^]^.

CT imaging remains the gold standard for diagnosing internal hernias, with sensitivity exceeding 82%. Key radiological findings to look for include dilated small bowel loops with transition points, whirling of mesenteric vessels, the presence of ascites or mesenteric vascular engorgement, and abnormal clustering of small bowel loops^[^4,12^]^.

Although leukocytosis is commonly associated with bowel ischemia and gangrene, a normal white blood cell count, as in our case, does not exclude these conditions. Laboratory findings in patients with strangulated bowel obstruction can be variable, and some patients with gangrenous bowel may initially present with normal or only mildly abnormal inflammatory markers. Therefore, clinical judgment remains paramount, and surgical intervention should not be delayed solely because the white blood cell count is within the normal range when there is a strong clinical suspicion of bowel ischemia or generalized peritonitis.

Regarding the preoperative evaluation, no additional imaging studies, including abdominal ultrasonography, plain abdominal radiography, or contrast-enhanced CT, were performed. The patient presented with clinical features of acute intestinal obstruction accompanied by generalized peritonitis, both of which constituted a clear indication for emergency exploratory laparotomy. Under these circumstances, further imaging would not have altered the need for urgent surgical intervention and could have resulted in unnecessary delay, potentially increasing the risk of bowel necrosis and other serious complications. In addition, contrast-enhanced CT was not available at our institution. Histopathological examination was not performed in this case because this service was unavailable at our hospital, and no nearby facility provided it. The available referral centers were located far from our institution, and the associated cost was also a limiting factor for the patient.

Previous reports of transmesoappendiceal hernia have described a variety of clinical presentations, hernia contents, and operative management strategies. Most reported cases, similar to our case, involved ileal herniation through the mesoappendiceal defect. The incarcerated bowel was successfully reduced in these cases, with defect management consisting mainly of appendectomy, with defect closure reported in one case. Some cases involved unusual hernia contents, such as Meckel’s diverticulum or the right ovary, demonstrating the variable nature of this internal hernia (Table 1).

**Table 1:** Previously reported cases of trans-mesoappendicular hernia in the English-language literature an overview.

Year	Age/sex	Author	Presentation	Diagnosis (hernia content)	Management
1963	80/M	JA Rooney^[^13^]^	SBO	Ileum	Bowel reduced, defect closed
2016	5/M	S Barman^[^14^]^	SBO	Meckel’s diverticulum	Content reduced, Resection and end-to-end anastomosis (REEA) of ileum, defect closed
2020	55/M	HG Vinay^[^15^]^	SBO	Ileum	Content reduced, appendectomy
2020	33/F	CE Jones^[^16^]^	Acute appendicitis	Right ovary	Ovary reduced, appendectomy (lap)
2024	50/M	EM Gashey^[^9^]^	SBO	Ileum	Ileum reduced, appendectomy
2025	76/F	RU Harvitkar^[^4^]^	SBO	Ileum	Ileum reduced, appendectomy
2025	79/F	P Gnanaselvam^[^11^]^	SBO	Ileum	Ileum reduced, appendectomy
2026	42/F	Our case	SBO	Ileum	Resection, ileostomy, appendectomy

Adapted from Gashey *et al*^[^9^]^ and updated with subsequently published cases.

Compared with previously reported cases, our patient had a more advanced presentation, characterized by strangulated small-bowel obstruction with a gangrenous ileum and appendiceal involvement, requiring bowel resection and formation of an ileostomy. In contrast, most previously described cases had viable bowel after reduction and did not require intestinal diversion. The decision to create an ileostomy in our case was due to an extensive fecal load in the colon and the need to avoid an unsafe primary anastomosis in an emergency setting.

The operative approach in previously reported cases has included both open and laparoscopic procedures, depending on the clinical condition and intraoperative findings. In our case, emergency exploratory laparotomy was performed because of the clinical features of complete SBO with generalized peritonitis. *En bloc* resection of the nonviable bowel and appendectomy were performed. These findings highlight the importance of individualized surgical decision-making based on bowel viability and the patient’s clinical condition rather than the anatomical defect alone.

Management of trans-mesoappendicular hernia is surgical and should be undertaken urgently once the diagnosis of SBO is established. The standard approach involves reduction of the herniated bowel and assessment of its viability if it is not frankly gangrenous, which requires *en bloc* resection, appendectomy, or closure or division of the defect to prevent recurrence^[^9,13^]^. Appendectomy is commonly performed, particularly when the appendix is involved in forming the defect or shows signs of inflammation, and both open and laparoscopic approaches have been described^[^4,9,11^]^.

This case adds to the limited body of literature on mesoappendiceal internal hernias and reinforces the importance of maintaining a high index of suspicion in patients presenting with SBO, particularly in the absence of common etiological factors. Early surgical exploration remains critical for both diagnosis and management of this potentially life-threatening condition.

This report describes a single case; therefore, the diagnostic findings, surgical decision-making, and management approach cannot be generalized to all patients with trans-mesoappendiceal internal hernia. Clinical presentation, intraoperative findings, bowel viability, and patient factors may vary considerably, requiring individualized assessment and treatment. In addition, the literature overview has inherent limitations because similar cases may have been reported using different anatomical or indexing terms. Therefore, the available published cases and the perceived rarity of this condition should be interpreted with caution.

## Conclusion

SBO secondary to internal herniation through a mesoappendiceal defect is an exceptionally rare surgical condition, but should be considered in the differential diagnosis in patients presenting with SBO. Timely recognition and prompt operative management are essential for favorable patient outcomes.

## Data Availability

The datasets used and analyzed during the current study are available from the corresponding author upon reasonable request.
